# Supplementary material for: Inside the black box: Refining programme theory in the PriDem dementia care study
Source: PLoS One. 2026 Mar 17;21(3):e0333154. doi: 10.1371/journal.pone.0333154 (PMC12995305; doi:10.1371/journal.pone.0333154)
Supplement: S1 Text — S1 File. PriDem Logic Model. S2 File. Standards for Reporting Qualitative Research (SRQR) Checklist. S3 File. Summary of evaluation findings already shared. S4 File. Adaptable PriDem resource pack with review templates. (ZIP) [file pone.0333154.s001.zip › S3 File - Summary of evaluation findings already shared.docx]

The study procedures and intervention were both feasible and acceptable, whilst QoL questionnaire ratings and service use saw no marked change from baseline to 9 months (26).

The proportion of personalised care plans significantly increased compared to preintervention during the intervention year, with an increased range of care domains discussed, demonstrating improved delivery of holistic care. CDL engagement and intervention flexibility enabled innovative multidisciplinary approaches to care planning, with PriDem templates adapted to local team needs [34].

In several practices, CDLs identified and engaged motivated staff, from care coordinators to GPs, who championed dementia care and fostered a sense of intervention ownership. This enabled tailored training, including joint visits and case discussions, leading to improved staff confidence and competence in post-diagnostic care. The process evaluation found that the flexibility of the intervention enabled some general practices to overcome the contextual barriers of financial constraints and primary care capacity, to innovate within the intervention goals, for instance developing multidisciplinary approaches to annual dementia reviews. CDLs engaged with key stakeholders to identify service gaps, with findings shared with commissioners to inform local pathway development . A PriDem service mapping template was created and shared with non-study regions. [35].
