# Supplementary material for: Inside the black box: Refining programme theory in the PriDem dementia care study
Source: PLoS One. 2026 Mar 17;21(3):e0333154. doi: 10.1371/journal.pone.0333154 (PMC12995305; doi:10.1371/journal.pone.0333154)
Supplement: S1 Text — S1 File. PriDem Logic Model. S2 File. Standards for Reporting Qualitative Research (SRQR) Checklist. S3 File. Summary of evaluation findings already shared. S4 File. Adaptable PriDem resource pack with review templates. (ZIP) [file pone.0333154.s001.zip › S4 File-Adaptable PriDem resource pack.docx]

**Annual dementia review: quick overview guide**

• Individuals have different [needs & priorities](#_Domain_3:_Patient/carer)—start by discussing the ‘[what could make a difference](#_What_Could_Make)’ document

• Remember to explore the **needs of the carer** as well as the patient—provide opportunities to speak in private

• Remember that patients & carers may not know which symptoms & problems are related to dementia

• Explore information needs & preferences throughout

• Explore patient’s [personal and social history](#_Domain_4:_Patient) to inform tailoring, including cultural/religious needs

• Discuss [services involved](#_Domain_1:_Services_1) & whether they are meeting current needs

• Add key actions to the care plan & use this to summarise the review for patients & carers

• **Click on each domain for more information & prompts if needed**

**PERSONALISED** [**CARE PLAN**](#_My_care_plan)

• Transfer agreed actions to care plan (specify time scale, responsibility)

• Review care plan with patient & carer. *On completion of review:* print copy for patient

& carer

**HOME ENVIRONMENT AND ADLs**

• Confirm living arrangements

• Explore how managing with household tasks & personal care

• Check for aids/adaptations & any safety concerns

• Check how managing finances & whether benefits check needed

• Check whether driving, whether DVLA informed & whether any concerns

**ACTIVITIES AND INTERESTS**

• Explore how patient & carer spend their days

• Explore opportunities for breaks from routine

• Check if maintaining or withdrawing from interests

• Check interest in taking part in dementia research

**DEMENTIA DIAGNOSIS & PROGRESSION**

*If first review since diagnosis*

• Explore feelings about diagnosis & if they have told people

• Check whether they have been offered CST, carer psychoeducation or other courses

*At all other reviews*

• Summarise key changes & explore information preferences regarding progression

• Consider whether the patient may be approaching end of life and should be put on palliative care pathway

**PSYCHOLOGICAL WELLBEING**

• Check for any cognitive changes (e.g. planning, concentration, word finding, multitasking)

• Explore new or upsetting behaviour changes (e.g. hallucinations, delusions)

• Check emotions/mood (e.g. apathy, low mood, overreacting) and sleep

• Check for social contact/isolation

• Observe relationship between patient & carer and explore any tensions

**MEDICATION REVIEW**

• Explore medication management & concordance

• Check understanding & concerns re: medications

• Check treatment targets are appropriate

• Check Anticholinergic Burden

*Dementia medications*

• Check anti-dementia medication is being prescribed if indicated

• Explore any side effects

• Consider whether second line drug should be added

**PHYSICAL HEALTH CHECK**

• Check height, weight, BP, immunisations, smoking, alcohol

• Explore sensory issues (eyes/hearing); mobility/falls; footcare; physical activity; dental care; continence; food/diet; pain/distress; swallowing; sialorrhoea; wounds/bruising; co-morbidities or investigations outstanding; infection delirium screen

• Observe for any communication difficulties (e.g. hesitancy, unclear speech, difficulty understanding)

• Review electronic frailty score/clinical frailty scale

• Check access to outpatients appointments/planned care

**SAFEGUARDING & ADVOCACY**

• Explore any concerns about safety at home or outside

• Explore causes (e.g. harassment, pressurised by cold callers, burglaries in area)

**PLANNING FOR CONTINGENCIES**

• Explore preferences for planning for contingencies

• Check if LPA for health/finance or deputyship

• Check for up to date DNACPR form

• Check for carer emergency card; carer back up plan; Herbert Protocol

• Consider other planning as appropriate (e.g. EHCP)

• Review & update any existing plans

**PROFESSIONAL NAMED POINT OF CONTACT**

• Check whether a named point of contact is recorded

• Explore whether/how named point of contact has been used

• Explore whether more intensive care co-ordination is needed

• Agree named point of contact/care co-ordinator going forward

© PriDem programme

Newcastle University & University College London 2022

Contents

[Guidance for review process 3](#_Toc96693364)

[Main domains of review 4](#_Toc96693373)

[Domain 1: Services currently involved 4](#_Toc96693374)

[Domain 2: Named point of contact 5](#_Toc96693375)

[Domain 3: Patient/carer goals and priorities 6](#_Toc96693376)

[Domain 4: Patient social and personal history to inform tailoring throughout 7](#_Toc96693377)

[Domain 5: Home environment and activities of daily living 8](#_Toc96693378)

[Domain 6: Activities and interests 9](#_Toc96693379)

[Domain 7: Safeguarding & advocacy 10](#_Toc96693380)

[Domain 8: Dementia diagnosis & progression 11](#_Toc96693381)

[Domain 9: Psychological wellbeing (cognitive, behavioural & emotional changes) 12](#_Toc96693382)

[Domain 10: Physical health check 13](#_Toc96693383)

[Domain 11: Medication review 16](#_Toc96693384)

[Domain 12: Planning for contingencies & changes 17](#_Toc96693385)

[Resources 18](#_Toc96693391)

[Domain 4: Patient social and personal history 18](#_Toc96693392)

[Domain 5: Home environment and activities of daily living 19](#_Toc96693395)

[Domain 6: Activities and interests 21](#_Toc96693399)

[Domain 7: Safeguarding & advocacy 22](#_Toc96693402)

[Domain 8: Dementia diagnosis and progression 23](#_Toc96693403)

[Domain 9: Psychological wellbeing (cognitive, emotional and behavioural changes) 25](#_Toc96693406)

[Domain 10: Physical health check 26](#_Toc96693409)

[Domain 11: Medication review 28](#_Toc96693412)

[Domain 12: Planning for contingencies and changes 29](#_Toc96693415)

[Information to be gathered at review 31](#_Toc96693386)

[References 32](#_Toc96693416)

Appendix 1: What could make a difference document……………………………………………………………………………………………..33

Appendix 2: Care plan template……………………………………………………………………………………………………………………………….34

# Guidance for review process

## **Preparations for the review**

Practical tasks to be completed and recorded **before the review** include: propose date(s) for review; send invitation letter to patient/carer; send what could make a difference document to patient/carer; confirm appointment(s)

It is recommended that practice teams consider how the tasks involved in the dementia review might be distributed to utilise the knowledge and skills of different practitioners.

### Preparing patients and carers

People with dementia and carers emphasise the value of face-to-face contact for the review, so where possible reviews should be conducted in person. For people with more advanced dementia, home visits may be more suitable.

To help prepare people with dementia and carers for the review and highlight concerns they would like to discuss, the ‘What could make a difference’ document can be used (provided as an [appendix](#_What_Could_Make_1) and as a separate document to allow customisation). This is designed to be sent to the patient and carer in advance of the review, and gives scope for each to consider what they would like to talk about either separately or together, so it is recommended that both the patient and carer are sent a copy.

## **Conducting the review**

An outline of basic information about the patient and carer (where relevant) that should be collated as part of the review is provided [at the end of this document](#_Information_to_be_1). Ideally patient and carer details will be populated automatically from their electronic patient record, and administrative staff will provide support with arranging the review and collating all agreed actions into a care plan.

### Facilitating involvement of PLWD and carers

During the review, it is important to provide opportunities for the person with dementia and their carer to talk separately wherever possible to enable them to discuss anything that they may have concerns about sharing when together.

### Safeguarding

Discussing different domains of the review may bring up different issues relevant to safeguarding. As part of any agreement to share responsibility for completing the review, all will need to be clear about their responsibilities in relation to safeguarding.

## **After the review**

Practical tasks to be completed and recorded **after the review** include: check that section on people involved in care plan is completed; collate all actions into care plan; send copy of care plan to patient/carer and professionals identified in [domain 1](#_Domain_1:_Services_1) (with patient’s consent).

### Care planning

It is important that all actions agreed as part of the review are collated in a care plan that is shared with the person with dementia and carer, and other professionals involved with their consent. A care plan template has also been developed for teams to use or help enhance current practice (provided as an [appendix](#_My_care_plan_1) and as a separate document to allow customisation).

[Return to overview](#_top)

# Main domains of review

## **Domain 1: Services currently involved**

| **Services for PATIENT** |
| --- |
| Who is the patient’s current named point of contact? |
| Does the patient have a care coordinator? |
| Is the patient under the care of social services?   - Social worker - Package of care (including number of visits per day, funding arrangements, whether a financial assessment has been carried out) |
|  |
| Under care of mental health services?   - Named psychiatrist - Under care of community mental health team - Under review by memory clinic |
|  |
|  |
| Under care of community services?   - E.g. community-based nurse, dietician, OT, physio, podiatrist, pharmacist |
| Details of other current services   - Home help/home care - Meals on wheels - Day centre - Telecare & assistive technology - Counselling service - Befriending - Anything else? |
|  |
|  |
|  |
|  |
|  |
|  |
| Services stopped with reason |
| Consent to share information & summary care record with other professionals) |
| **Services for CARER** |
| Are you aware of/in touch with carer services? |
| Have you had a carer assessment? If not, explore interest and provide information |
| Have you completed a carer education programme? If not, explain what is involved, explore interest and provide information |

[Return to overview](#_top)

## **Domain 2: Named point of contact**

| **Prompts** |
| --- |
| How are you managing to keep track of different services involved in your care? |
| How much have you been in touch with your named point of contact over the last year?  How helpful have they been able in answering all of your queries? |
| *Record recommendation re named point of contact on care plan*  *NB named point of contact could be an individual or a team* |

[Return to overview](#_top)

## **Domain 3: Patient/carer goals and priorities**

| **Prompts** |
| --- |
| - What do you want to talk about today?   - *Also see ‘What could make a difference’ sheet (if completed beforehand)* |
| - What is going well at the moment? - What isn’t going so well? |
| - What are the key changes that you have noticed in the last year?   *E.g., changes in memory; orientation (time, place, person); problem solving & judgement; independence in ADL; hobbies & interests; self-care & continence; personality and emotional behaviour; sleep)* |
| - Is there any information you need at the moment? - *Explore current information needs of patient and carer*   - *Topics (as issues are discussed throughout the review, check for additional information needs)*   - *Preferred format (e.g., written, telephone, discussion, web resources including blogs and videos, individual vs group)* |

[Return to overview](#_top)

## **Domain 4: Patient social and personal history to inform tailoring throughout**

| **Initial prompts** | **Prompts for gathering more detail** |
| --- | --- |
| - It is helpful to know about you to help us tailor your care and support. Can you tell me about important things in your life? - *These topics may also be conversationally explored during the review* | - What is important now? e.g social activity, what they enjoy doing, spiritual and/or cultural needs, traditions, routines, preferences and values. - What has been significant in your life? e.g important experiences or events, roles, accomplishments, losses/bereavements, background, and work history. - Sometimes people have ideas about what they would like to happen in their future, like things they would like to do or places they would like to live. Do you have any plans or wishes for the future? |

[Further resources](#_Domain_4:_Patient_1) | [Return to overview](#_top)

## **Domain 5: Home environment and activities of daily living**

| **Initial prompts** | **Prompts for gathering more detail** |
| --- | --- |
| - Do you live alone or with others? |  |
| - How are you managing with running your home at the moment? - Do you have any adaptations or aids at home to help? | - Do you have smoke & carbon monoxide alarms? - Have these alarms been checked recently? |
|  | - How are you getting on with home maintenance? |
|  | - Are you keeping warm/cool enough? |
|  | - How are you managing with keeping things clean and tidy? |
|  | - Have you had any modifications to help (e.g. wetroom, stair lift, hand rails, grab rail, ramps, automatic lighting)? |
|  | - Do you have telecare (e.g. remote monitored pendant alarm or sensor pads)? |
| - How are you managing with day to day life? - Thinking about a typical day, are there activities that are getting a bit more difficult or that you need a bit of help with? | - *Explore any difficulties with* - *getting dressed and undressed* - *using the toilet (including getting up)* - *making meals (including burning food, injuries)* - *eating and drinking* - *shopping* - *using the telephone/a mobile phone* - *getting around (walking indoors and outdoors, using stairs, getting in/out of bed, getting in/out of chair, getting in/out of car, public transport, visiting shops/banks/post office etc)* |
| - How are you managing with money? | *Explore any difficulties with*   - *day to day purchases* - *dealing with bills* - *managing paperwork* - *accessing money (e.g. cashpoint, online banking, cheque)* |
| - A number of benefits may be available to you (PLWD and carer). There is also practical support available to make applications for benefits. Is this something you would like to explore further? |  |
| **Driving** | |
| - Are you driving?   **IF YES:**   - Have you had any driving accidents or near misses? Any little scrapes or bumps in the car? - Have any of your family expressed concerns about your driving? - Have you got lost driving in a familiar area or lost your car?   **ASK CARER:** Do you have any concerns about their driving? |  |
| **Initial prompts for CARER** | |
| - How are you managing with day to day activities at the moment? - Are there any tasks that you are struggling with? |  |

[Further resources](#_Domain_5:_Home_1) | [Return to overview](#_top)

## **Domain 6: Activities and interests**

| **Initial prompts** | **Prompts for gathering more detail** |
| --- | --- |
| - How do you spend your days? - What kinds of activities, interests of hobbies do you do? | - *Explore* - *what matters to the person – their priorities, interests, values and motivations (see also Social and Personal history)* - *any groups and services the person is connected to or has previously been* - *what the person can do for themselves, in order to keep well and active* - *things they used to like to do but no longer do* - *assets people already have that they can draw on – family, friends, hobbies, skills and passions* |
| - Are you finding it harder to take part in activities you enjoy? | - *Explore potential barriers such as transport, motivation, confidence, fear of falling, cost, lack of information* |
| - Have you been able to have any breaks from routine (e.g. holidays or days out)? |  |
| - Are you interested in taking part in dementia research? *(If yes, refer to Join Dementia Research)* |  |

[Further resources](#_Domain_6:_Activities_2) | [Return to overview](#_top)

## **Domain 7: Safeguarding & advocacy**

| **Initial prompts** | **Prompts for gathering more detail** |
| --- | --- |
| *Separate patient and career to ask about any concerns you have picked up during consultation (e.g. say “I am just going to ask carer to leave the room while I examine you/go with you to collect a urine sample etc.)*   - We routinely ask everybody because this is a safe space for you to express your worries: do you feel safe from harm or pressure from other people? - Do you feel safe in your home? - Do you feel safe when you go out? | - *Explore causes of feeling unsafe e.g. harassment, pressurised by cold callers or doorstep sellers, burglaries in area* |

[Further resources](#_Domain_7:_Safeguarding_3) | [Return to overview](#_top)

## **Domain 8: Dementia diagnosis & progression**

**AT FIRST REVIEW FOLLOWING DIAGNOSIS**

| **Initial prompts** |
| --- |
| - How did it go at the memory clinic and what did they tell you? - How are you feeling about this? - Have you been able to tell people important to you about your diagnosis? |
| - I appreciate that when you get a diagnosis it can be hard to take things in. Now you have had a bit of time, is there anything you would like to ask me about your diagnosis? |
| - Has anyone talked to you about any short courses for people where you can learn practical tips and strategies to help you to cope with the changes in your memory and thinking? |

**AT ALL OTHER REVIEWS**

| **Initial prompts** |
| --- |
| - *Summarise key changes* - *Explore information preferences regarding progression* |
| *Consider whether the patient may be approaching end of life and should be put on the palliative care pathway*  *IF SO: Use palliative care template and consider whether EHCP is needed* |

[Further resources](#_Domain_8:_Dementia_1) | [Return to overview](#_top)

## **Domain 9: Psychological wellbeing (cognitive, behavioural & emotional changes)**

| **Initial prompts** | **Prompts for gathering more detail** |
| --- | --- |
| - What are the main changes that you have noticed in the last year?   *NB: Probe for each specific area below since patients & carers may not attribute these changes to dementia.* |  |
| **Cognitive changes** | |
| - Have you noticed any changes in your memory or thinking abilities (e.g. concentration, word finding, planning or multitasking)? |  |
| **Behavioural changes** | |
| - Have there been any new or upsetting behaviours? | *Common new behaviours include wandering, psychomotor agitation, verbal or physical aggression* |
| **Hallucinations** | |
| - Have you ever seen or heard anything that others say aren’t there? |  |
| **Emotions and mood** | |
| - How is your mood? - Do you ever feel particularly worried or stressed? - Have you noticed any changes in your confidence or motivation? - Have there been changes in your emotional responses, for example, being more irritable, having sudden mood changes or overreacting to things? - Have you lost interest in things that you used to enjoy? |  |
| **Sleep** | |
| - Do you have trouble sleeping?   **Ask the carer**   - How do any sleep problems affect you? |  |
| **Social contact/isolation and relationships** | |
| - Do you speak to or see family & friends as often as you like to? - How are you getting on with each other (ask PLWD and carer separately if possible)? |  |
| **Initial prompts for CARER** | |
| - How are you coping with these changes? - Explore information needs about changes and progression |  |

[[Further resources](#_Domain_9:_Psychological_1) | Return to overview](#_top)

## **Domain 10: Physical health check**

| **Initial prompts** | **Prompts for gathering more detail** |
| --- | --- |
| - *BP (lying and standing)* - *Height (if not measured in the last 5 years)* - *Weight/BMI NB: If not possible to weigh the patient, ask about any recent weight change (e.g. do your clothes feel tighter, looser or about the same?)* |  |
| **Sensory issues** | |
| - *When eyes were last tested* - *Any issues with sight (including use of glasses)* - *Last hearing test* - *Any problems with hearing (including use of hearing aids)* | - Do you wear your glasses? (3Cs – correct, clean & current glasses) - Do you wear your hearing aid? - Has the carer noticed any changes that may indicate vision problems (e.g. constantly cleaning glasses, falls, disinterest in hobbies) - Has the carer noticed any changes that may indicate hearing problems (e.g. avoiding social events, becoming withdrawn or frustrated, increasing the volume on the TV))   [Further resources](#_Sensory_needs_(Return) |
| **Mobility/getting around** | |
| - Do you have any difficulty walking? - Do you have any difficulty going up or down stairs? - Have you fallen in the last year (Including slips or trips) in which you lost your balance and landed on the floor, the ground or a lower level? | - *Consider balance, gait and mobility* - *Observe mobility* - Do you use any aids to help you move around (e.g. walking stick, walker, rollator, wheelchair)? - Do you worry about falling? (check patient and carer perspectives) - *Check for a reluctance to go out, or becoming more sedentary*   [Further resources](#_Mobility_/_falls) |
| **Foot care** | |
| - Can you cut your own toenails? - Do you have any problems with your feet? | [Further resources](#_Footcare_(Return_to) |
| **Physical activity** | |
| - What do you do to keep active? | - *Explore walking, swimming, strength training, flexibility, balance*   [Further resources](#_Physical_activity_(Return) |
| **Dental care** | |
| - Have you seen a dentist in the last year? - Do you have any pain or problems with your teeth (or dentures)? - Do you have any problems cleaning your teeth? | [Further resources](#_Dental_care_(Return) |
| **Continence** | |
| - How are you managing to use the toilet at the moment? - Do you experience any problems controlling your bladder or bowels? - Do you suffer from constipation? | [Further resources](#_Continence_(Return_to) |
| **Smoking** | |
| - Do you smoke or use any form of tobacco? - How many do you smoke on average daily? - Would you like support to stop smoking? | [Further resources](#_Smoking_(Return_to) |
| **Alcohol** | |
| - Do you drink alcohol? - How often do you have a drink containing alcohol? - How many units of alcohol do you drink on a typical day when you are drinking? - How often have you had 6 or more units if female, or 8 or more if male, on a single occasion in the last year? - Would you like support to stop or reduce your alcohol intake? | [Further resources](#_For_PLWD_&) |
| **Food/diet** | |
| - Do you have a good appetite? - Has your appetite changed recently? - How many cups of fluid do you have each day? | [Further resources](#_Food_/_diet) |
| **Immunisations** | |
| - *Check immunisations record* | *Check flu vaccine; COVID-19 (check number of doses, booster); Tetanus; Pneumonia for PLWD and carer (if registered)* |
| **Communication** | |
| *Observe for the following signs of difficulties:*   - *Difficulty recalling the right words* - *Unclear speech; difficulty articulating speech sounds* - *marked hesitancy when speaking; speech is not fluent/free flowing* - *alternatively, fast free-flowing speech, with the person having little awareness that they are not being understood (common in ‘Semantic Dementia’)* - *increasing difficulty understanding what other people are saying, especially when those other people are speaking very fast* *and/or using long and/or complex utterances* - *significant reliance on the carer to communicate for the person* - *Carer or PWD frustration about communication issues* | [Further resources](#_For_PLWD_&) |
| **Pain/distress** | |
| - Do you have any pain anywhere? | - *Observable signs of pain may include:* - *breathing: laboured breathing or hyperventilating* - *vocalization: moaning or crying* - *facial expression: frowning or grimacing* - *body language: clenching fists or pushing away caregivers* - *inconsolable* - *If unable to verbalise or there is need to re-assess pain for pwd e.g following a pain management intervention, consider using a structured observation tool* - *There is no evidence to recommend any specific measure, one option is the Abbey Pain Scale:* <https://prc.coh.org/PainNOA/Abbey_Tool.pdf> |
| **Swallowing** | |
| - Do you have any difficulties with swallowing? | - *Observable signs of swallowing difficulties include:* - *Repeated bouts of chest infections or pneumonia* - *Repeated coughing, throat clearing or choking after swallowing food or drink* - *A wet sounding voice after swallowing* - *Expressing fear during eating* - *A reluctance to eat and drink* - *Holding food in the mouth* - *Packing food into the cheeks* - *Swallowing several times on one bite* - *Grimacing when swallowing* - *Tilting the head back to eat and drink* - *Food or liquid falling out of the mouth or drooling liquids from the mouth* - *Exaggerated movements of the jaw, lips or tongue* - *A delay in swallowing after the food has been chewed* - *Tiredness during or after a meal* - *Weight loss over time*   [Further resources](#_Swallowing_(Return_to) |
| **Sialorrhoea** | |
| - Do you have any problems with excess saliva or drooling? |  |
| **Skin** | |
| - *Is skin intact?* - *Are there pressure ulcers, moisture lesions or other wounds?* - *Is there bruising/rashes?* |  |
| Co-morbidities | |
| - *Check for any outstanding investigations* |  |
| Infection delirium screen | |
| - *Screen for UTI* |  |
| **Frailty** | |
| - *Review electronic frailty score or consider completing the clinical frailty scale* | *Clinical frailty scale:*  <https://www.bgs.org.uk/sites/default/files/content/attachment/2018-07-05/rockwood_cfs.pdf> |
| **Access to outpatients appointments and planned care** | |
| - How are you managing with your appointments? | *Prompt for remembering appointments, access issues* |

[Further resources](#_Domain_10:_Physical_1) | [Return to overview](#_top)

## **Domain 11: Medication review**

| **Initial prompts** | **Prompts for gathering further detail** |
| --- | --- |
| - How are you managing your medications? - Please tell me/show me any medications you don’t like to take? Why is this? - What would help you to manage your medication? | - *Probe for any assistance needed, frequency of missed doses (ask about extra packs of medication stored at home), whether any aids are used (and how successful these are). Consider blister pack or automated devices.* - *Consider any safety issues* |
| **Dementia medication** | |
| - Is the pwd currently prescribed anti-dementia medication? - IF YES: Are there any side effects? Consider whether there is any indication for adding a second line drug (e.g. memantine)? - IF NO: Consider whether the patient is eligible? |  |
| **Medication review** | |
| - *Explore views and understanding about current medicines with patient & carer* - *Explore any concerns, questions or problems with the medicines with patient & carer* - Are you taking any other medications not issued by the doctor (i.e. over-the-counter and complementary medicines)? - *Explore how well current medications work for the person, how appropriate they are, and whether their use is in line with national guidance* - *Consider whether the person has had or has any risk factors for developing adverse drug reactions (report adverse drug reactions in line with the yellow card scheme)* - *Check treatment targets are appropriate (e.g. blood pressure, diabetes)* | - *Consider STOPP-START CGA toolkit Plus if over 65 years*   <https://www.cgakit.com/m-2-stopp-start> |
| *Use the Anticholinergic Cognitive Burden to identify medications which may have adverse cognitive effects* | *Anticholinergic burden score (can either enter all current medications or look at alphabetical list of* *common medications to calculate the score). It also provides advice on reducing risk:*  <http://www.acbcalc.com/> |

[Further resources](#_Domain_11:_Medication_1) | [Return to overview](#_top)

## **Domain 12: Planning for contingencies & changes**

| **Initial prompts** | **Prompts for further exploration** |
| --- | --- |
| - Sometimes people have ideas about what they would like to happen in their future, like things they would like to do or places they would like to live. Do you have any plans or wishes for the future? | - It’s often helpful to have thought through different situations in advance, to avoid having to make rapid decisions at a time when you may be feeling unwell or even unconscious. Even planning for simple things such as who is going to feed your pet if you get poorly can avoid stress at a later stage? - What about your healthcare? It is sometimes helpful to have thought about decisions in advance so that we know what sorts of care you would like to receive in case you are too poorly to tell us at the time. The sorts of things you can think about in advance are treatments you would or wouldn’t want to be given. - Thinking further ahead for the future, some people find it reassuring to plan towards the end of their life. For example, telling others where they might want to be cared for in their last days or religious wishes they might have. |
| - Some people find it reassuring to sort out their affairs and make a Power of Attorney or Will. Do you have a Lasting Power of Attorney for health and welfare? |  |
| - Do you have a Lasting Power of Attorney for property and finance? |  |
| *Is there an up to date DNACPR form or ADRT in the notes?* | *IF NOT: start a conversation. See* [*Resources*](#_Resources:_Domain_12:) *for guidance.* |
| **Initial prompts for CARER** | |
| - There are things that can be put in place to help in the event of an emergency. Do you have   - a carer back up plan?   - a carer emergency card?   - any other plans for emergencies in place (e.g. ‘Message in a Bottle’ for paramedics)? |  |
| Have you completed the Herbert protocol (if the PWD is at risk of going missing)? |  |

[Further resources](#_Domain_12:_Planning_1) | [Return to overview](#_top)

## **Resources: Domain 4: Patient social and personal history**

| **For PLWD & carers** | **For professionals** |
| --- | --- |
| ‘This is me’ template to help collate information about the person: <https://www.alzheimers.org.uk/get-support/publications-factsheets/this-is-me> |  |

[Return to Domain 4](#_Domain_4:_Patient) | [Return to overview](#_top)

## **Resources: Domain 5: Home environment and activities of daily living**

| **For PLWD & carers** | **For professionals** |
| --- | --- |
| **Fire safety** | |
| To check eligibility for a safe and well check in Newcastle: <https://www.twfire.gov.uk/safety-advice/home/home-safety-checks/> To check eligibility for a safe and well check in Barnet: <https://www.london-fire.gov.uk/safety/the-home/home-fire-safety-visits/> |  |
| **Managing in warm / cold weather** | |
| Age UK Warm Homes programme: <https://www.ageuk.org.uk/services/in-your-area/warm-homes/>  Advice on managing in hot weather: <https://www.dementiauk.org/keeping-a-person-with-dementia-safe-during-hot-weather/>  Advice on managing in cold weather:  <https://www.alzheimers.org.uk/get-support/daily-living/keeping-warm-dementia> |  |
| **Home safety & maintenance** | |
| Advice on making the home safe: <https://www.dementiauk.org/get-support/maintaining-health-in-dementia/safe-comfortable-home-for-a-person-with-dementia/>  Age UK handyperson service: <https://www.ageuk.org.uk/services/in-your-area/handyperson-services/> |  |
| **Home adaptations** | |
| Information about home adaptations assessment: <https://www.nhs.uk/conditions/social-care-and-support-guide/care-services-equipment-and-care-homes/home-adaptations/> |  |
| **Accessible toilets and disabled parking** | |
| To buy a RADAR key for accessible toilets: <https://www.disabilityrightsuk.org/shop/official-and-only-genuine-radar-key>  To apply for a blue badge for disabled parking: <https://www.alzheimers.org.uk/blog/how-apply-blue-badge-person-dementia>v |  |
| **Managing money & financial support** | |
| Advice for PLWD on managing money:  <https://www.alzheimers.org.uk/sites/default/files/2020-01/1501LP%20-%20managing%20your%20money.pdf>  Benefits for PLWD and carers:  <https://www.dementiauk.org/get-support/legal-and-financial-information/getting-financial-help-and-support/>  Carer benefits:  <https://www.nhs.uk/conditions/social-care-and-support-guide/support-and-benefits-for-carers/benefits-for-carers/>  Benefits for people of working age:  <https://www.alzheimers.org.uk/get-support/legal-financial/benefits-working-age>  Benefits calculator:  <https://www.entitledto.co.uk/benefits-calculator/Intro/Home?cid=717a8dbe-65f6-46dd-8dc8-0565caf91507> |  |
| **Driving** | |
| Advice for PLWD:  <https://www.alzheimers.org.uk/sites/default/files/2018-10/AS_NEW_Living%20with%20Dementia_Driving_ONLINE.pdf>  Advice for carers/family:  <https://www.alzheimers.org.uk/sites/default/files/2018-10/439LP%20Driving%20and%20dementia.pdf> | [https://research.ncl.ac.uk/driving-and-dementia/ consensusguidelinesforclinicians/Final%20Guideline.pdf](https://research.ncl.ac.uk/driving-and-dementia/consensusguidelinesforclinicians/Final%20Guideline.pdf) |

[Return to Domain 5](#_Domain_5:_Home) | [Return to overview](#_top)

## **Resources: Domain 6: Activities and interests**

| **For PLWD & carers** | **For professionals** |
| --- | --- |
| General information and activity ideas: <https://www.nhs.uk/conditions/dementia/activities/>  Information about reminiscence therapy: <https://www.alzheimers.org.uk/categories/treatments-and-therapies/reminiscence-therapy> | Tool for guiding conversations about physical activity with PLWD: <https://movingmedicine.ac.uk/consultation-guides/condition/adult/dementia/> |
| **Specific resources for marginalised groups** | |
| Dementia friendly faith communities: <https://www.alzheimers.org.uk/get-involved/dementia-friendly-communities/faith-groups>  LGBT+: [https://www.alzheimers.org.uk/sites/default/files/pdf/ lgbt_living_with_dementia.pdf](https://www.alzheimers.org.uk/sites/default/files/pdf/lgbt_living_with_dementia.pdf)  BME communities:  <https://www.scie.org.uk/dementia/living-with-dementia/bme/>  Veterans with dementia:  <https://www.britishlegion.org.uk/stories/how-we-support-veterans-with-dementia> |  |
| **If interested in taking part in research** | |
| <https://www/joindementiaresearch.nihr.ac.uk/> |  |

[Return to Domain 6](#_Domain_6:_Activities) | [Return to overview](#_top)

## **Resources: Domain 7:** **Safeguarding & advocacy**

| **For PLWD & carers** | **For professionals** |
| --- | --- |
| General information about safeguarding: <https://www.nhs.uk/conditions/social-care-and-support-guide/help-from-social-services-and-charities/abuse-and-neglect-vulnerable-adults/> |  |
| General information about advocacy: <https://www.nhs.uk/conditions/social-care-and-support-guide/help-from-social-services-and-charities/someone-to-speak-up-for-you-advocate/> |  |
| The Herbert Protocol – specific guidance where the person getting lost is a concern: <https://www.ageuk.org.uk/calderdaleandkirklees/about-us/latest-news/articles/2018/the-herbert-protocol/> |  |

[Return to Domain 7](#_Domain_7:_Safeguarding_3) | [Return to overview](#_top)

## **Resources: Domain 8: Dementia diagnosis and progression**

| **For PLWD & carers** | **For professionals** |
| --- | --- |
| **General information about dementia** | |
| ‘The brain tour’ provides information about dementia using a picture of the different areas of the brain and explains how they link to dementia subtypes: <https://www.alzheimersresearchuk.org/dementia-information/need-to-know-more/the-brain-tour/>  ‘A walk through dementia’ is an app designed to put you in the shoes of someone living with dementia from Alzheimer’s Research UK  <https://www.awalkthroughdementia.org/> |  |
| **Learning disability & dementia** | |
| Information & advice for carers:  [https://www.alzheimers.org.uk/about-dementia/types-dementia/learning-disabilities-dementia-treatments#content-start](https://www.alzheimers.org.uk/about-dementia/types-dementia/learning-disabilities-dementia-treatments%23content-start)  <https://www.dementiauk.org/get-support/maintaining-health-in-dementia/learning-disability-and-dementia/> |  |
| **Alzheimer’s disease** | |
| <https://www.alzheimersresearchuk.org/dementia-information/types-of-dementia/alzheimers-disease/> |  |
| **Dementia with Lewy bodies and dementia in Parkinson’s disease** | |
| The Lewy Body Society includes a guide to LBD, videos, blogs and leaflets (including one on managing hallucinations):  <https://www.lewybody.org/> | A management toolkit for professionals with a one-page overview of the major symptoms in LBD and their management: <https://research.ncl.ac.uk/diamondlewy/managementtoolkit/> |
| **Frontal lobe dementia, Pick’s disease, progressive aphasia** | |
| Rare dementia support includes videos, information, advice, details of UK meetings: <https://www.raredementiasupport.org/frontotemporal-dementia/> |  |
| **Vascular dementia** | |
| An illustration of what it is like for one person living with vascular dementia and links to additional information is available from: <https://www.bhf.org.uk/informationsupport/heart-matters-magazine/medical/living-with-vascular-dementia-diane-story> |  |
| **Young onset dementia** | |
| Information and personal stories: <https://www.dementiauk.org/about-dementia/young-onset-dementia/what-is-young-onset-dementia/>  Information sheet: <https://www.alzheimers.org.uk/about-dementia/types-dementia/younger-people-with-dementia?documentID=164> | Videos about the needs of people with young onset dementia and their family members: <https://www.youngdementianetwork.org/resources/good-practice-guide/key-needs-videos/>  <https://www.scie.org.uk/dementia/symptoms/young-onset/living-with-young-onset-dementia-video.asp> |

[Return to Domain 8](#_Domain_8:_Dementia) | [Return to overview](#_top)

## **Resources: Domain 9: Psychological wellbeing (****cognitive, emotional and behavioural changes)**

| **For PLWD & carers** | **For professionals** |
| --- | --- |
| **Specific resources relating to carers** | |
| Resource with text and videos: <https://healthtalk.org/carers-people-dementia/advice-to-other-carers>  Carer checklist: <https://www.ageuk.org.uk/globalassets/age-uk/documents/information-guides/age-uk-carers-checklist.pdf>  Information for carers about looking after themselves:  <https://www.dementiauk.org/get-support/looking-after-yourself-as-a-carer/looking-after-yourself-when-you-care-for-someone-with-dementia/> | **Carer psychoeducation courses:**  START is a manualised one-to-one intervention. Although it is not available in all locations, carer manuals are available online in English, Hindi, Urdu, Japanese and Spanish. After selecting the language, scroll down to Carer Version: <https://www.ucl.ac.uk/psychiatry/research/mental-health-older-people/projects/start-resources/start-manuals>  The Carer Information and Support Programme (CrISP) is a group psychoeducation course for carers, but may not be available in all areas and availability may be limited by COVID-19 restrictions: <https://www.alzheimers.org.uk/about-us/our-dementia-programmes/carer-information-support-programme> |
| **Sleep**  Advice for PLWD: [https://www.dementiauk.org/wp-content/uploads/2019/ 06/good-habits-for-bedtime-new-web.pdf](https://www.dementiauk.org/wp-content/uploads/2019/%2006/good-habits-for-bedtime-new-web.pdf)  Advice for carers: <https://www.dementiauk.org/sleep-deprivation-dementia/> |  |

[Return to Domain 9](#_Domain_9:_Psychological) | [Return to overview](#_top)

## **Resources: Domain 10: Physical health check**

| **For PLWD & carers** | **For professionals** |
| --- | --- |
| Alcohol ([Return to domain 10](#_Domain_10:_Physical) \| [Return to overview](#_top)) | |
| Advice on drinking: <https://www.wearewithyou.org.uk/help-and-advice/>  Find local services through:  <https://www.nhs.uk/live-well/alcohol-support/> |  |
| Communication ([Return to domain 10](#_Domain_10:_Physical) \| [Return to overview](#_top)) | |
|  | Information on the support Speech and Language Therapists can offer: <https://www.rcslt.org/wp-content/uploads/media/Project/RCSLT/rcslt-dementia-factsheet.pdf> |
| Continence ([Return to domain 10](#_Domain_10:_Physical) \| [Return to overview](#_top)) | |
| General information:  <https://www.alzheimers.org.uk/sites/default/files/migrate/downloads/factsheet_continence_and_using_the_toilet.pdf>  <https://www.dementiauk.org/wp-content/uploads/2019/05/continence-new-style-WEB.pdf>  Information and advice on reducing risk of UTIs is available from: <https://www.alzheimers.org.uk/get-support/daily-living/urinary-tract-infections-utis-dementia> |  |
| Dental care ([Return to domain 10](#_Domain_10:_Physical) \| [Return to overview](#_top)) | |
| General advice on mouth care: <https://www.dementiauk.org/mouth-care/>  Finding a dentist: [https://www.alzheimers.org.uk/get-support/daily-living/finding-nhs-dentist#content-start](https://eur03.safelinks.protection.outlook.com/?url=https%3A%2F%2Fwww.alzheimers.org.uk%2Fget-support%2Fdaily-living%2Ffinding-nhs-dentist%23content-start&data=04%7C01%7Cclaire.bamford%40newcastle.ac.uk%7C431d399de55c4303269808d99efeca92%7C9c5012c9b61644c2a91766814fbe3e87%7C1%7C0%7C637715639715161424%7CUnknown%7CTWFpbGZsb3d8eyJWIjoiMC4wLjAwMDAiLCJQIjoiV2luMzIiLCJBTiI6Ik1haWwiLCJXVCI6Mn0%3D%7C1000&sdata=wkpXLhCDLvtzXJoXRqB3ZUSgHkchLGzgkcnBAvQWzhQ%3D&reserved=0) |  |
| Food / diet ([Return to domain 10](#_Domain_10:_Physical) \| [Return to overview](#_top)) | |
| Information sheets:  [https://www.dementiauk.org/wp-content/uploads/2021/03/ DUKFS17_Eating_Drinking-2021_March2021.pdf](https://www.dementiauk.org/wp-content/uploads/2021/03/DUKFS17_Eating_Drinking-2021_March2021.pdf)  <https://nhsforthvalley.com/wp-content/uploads/2015/06/Nutrition-and-Dementia.pdf>  Video of a person with dementia talking about her experience of changes including changes in taste: <https://www.youtube.com/watch?v=VH_QpmH_lhU> | If considering enteral feeding for people with severe dementia, use the NICE decision aid:  <https://www.nice.org.uk/guidance/ng97/resources/enteral-tube-feeding-for-people-living-with-severe-dementia-patient-decision-aid-pdf-4852697007> |
| Footcare ([Return to domain 10](#_Domain_10:_Physical) \| [Return to overview](#_top)) | |
| General advice: <https://www.ageuk.org.uk/information-advice/health-wellbeing/exercise/fitter-feet/>  Help with cutting toenails may be available through Age UK: <https://www.ageuk.org.uk/services/in-your-area/foot-care/> |  |
| Mobility / falls ([Return to domain 10](#_Domain_10:_Physical) \| [Return to overview](#_top)) | |
| Advice on falls and dementia: <https://www.nhsinform.scot/healthy-living/preventing-falls/falls-and-dementia> |  |
| Physical activity ([Return to domain 10](#_Domain_10:_Physical) \| [Return to overview](#_top)) | |
| General advice on staying healthy: <https://www.dementiauk.org/wp-content/uploads/2020/08/Staying-healthy-1.pdf> | Suggestions for how to start a conversation about physical activity are available at: <https://movingmedicine.ac.uk/consultation-guides/condition/adult/dementia/#start> |
| Sensory needs ([Return to domain 10](#_Domain_10:_Physical) \| [Return to overview](#_top)) | |
| Information about sensory loss: <https://www.alzheimers.org.uk/about-dementia/symptoms-and-diagnosis/sight-hearing-loss>  Video of a person with dementia talking about her experience of sensory changes & accompanying leaflet: <https://www.youtube.com/watch?v=VH_QpmH_lhU>  <https://www.lifechangestrust.org.uk/sites/default/files/Leaflet.pdf> | Recommendations & strategies for assessing vision and hearing in PLWD: <https://bmjopen.bmj.com/content/8/1/e019451> |
| Smoking ([Return to domain 10](#_Domain_10:_Physical) \| [Return to overview](#_top)) | |
| Advice on smoking: <https://www.alzheimers.org.uk/get-support/daily-living/smoking-and-alcohol-dementia> |  |
| Swallowing ([Return to domain 10](#_Domain_10:_Physical) \| [Return to overview](#_top)) | |
| Detailed guide about swallowing problems in dementia: [https://www.uhcw.nhs.uk/clientfiles/files/ Dementia%20Care%20Swallowing%201807%20April%2014.pdf](https://www.uhcw.nhs.uk/clientfiles/files/Dementia%20Care%20Swallowing%201807%20April%2014.pdf) | *If considering enteral feeding for people with severe dementia, use the NICE decision aid:*  <https://www.nice.org.uk/guidance/ng97/resources/enteral-tube-feeding-for-people-living-with-severe-dementia-patient-decision-aid-pdf-4852697007> |

##

## **Resources: Domain 11: Medication review**

| **For PLWD & carers** | **For professionals** |
| --- | --- |
|  | Advice for clinicians on prescribing acetylecholinesterase inhibitors: <https://www.sunderlandccg.nhs.uk/wp-content/uploads/2020/07/Acetylcholinesterase-information-leaflet.pdf> |
|  | Advice for GPs on prescribing memantine: <https://www.england.nhs.uk/london/wp-content/uploads/sites/8/2019/07/dem-memantine-dec18.pdf> |
|  | NICE guidance on use of anti-psychotics in dementia:  <https://www.nice.org.uk/guidance/ng97/resources/antipsychotic-medicines-for-treating-agitation-aggression-and-distress-in-people-living-with-dementia-patient-decision-aid-pdf-4852697005> |
|  | Anticholinergic burden score (can either enter all current medications or look at alphabetical list of common medications to calculate the score). Also provides advice on reducing risk:  <http://www.acbcalc.com/> |

[Return to Domain](#_Domain_11:_Medication) 11 | [Return to overview](#_top)

## **Resources: Domain 12:** **Planning for contingencies and changes**

| **For PLWD & carers** | **For professionals** |
| --- | --- |
| **Advance care planning** | |
| Deciding Right resources for patients and the public: <https://northerncanceralliance.nhs.uk/deciding-right/deciding-right-information-for-patients-and-public/> | The Deciding Right website contains educational resources for professionals; a workbook; presentations; and a linked app):  <https://northerncanceralliance.nhs.uk/deciding-right/>  Gold standards framework:  <https://www.goldstandardsframework.org.uk/advance-care-planning>  There is a framework to facilitate discussions of ACP by telephone at: <https://compassionindying.org.uk/library/advance-care-planning-by-phone-or-video/> |
| **Lasting Power of Attorney** | |
| Guide and forms available from: <https://www.gov.uk/government/publications/make-a-lasting-power-of-attorney>  Information on legal issues:  <https://www.dementiauk.org/get-support/legal-and-financial-information/sources-of-support-and-advice/> |  |
| **DNACPR** | |
|  | 15 minute worksheet on issues around resuscitation available from: <https://northerncanceralliance.nhs.uk/wp-content/uploads/2018/11/CLIP-Issues-around-Resusciatation.pdf>  Guide to having DNACPR conversations: <https://geekymedics.com/dnacpr-discussions-and-documentation/>  Forms available from Deciding Right: <https://northerncanceralliance.nhs.uk/wp-content/uploads/2018/11/DNACPR-NHS-Fillable-form-v17.pdf> |
| **Carer planning for emergencies** | |
| Carer back up plan - online form from CarersUK at:  <https://carersdigital.org/mybackup/>  Emergency carers card scheme (Newcastle): <https://www.informationnow.org.uk/organisation/newcastle-emergency-carers-card-scheme/>  Emergency carers card scheme (Brent): <https://brentcarerscentre.org.uk/resources/downloads-carers/emergency-planning/>  The Herbert Protocol if concerns about the person with dementia going missing - form and further information, including video available from: <https://www.westyorkshire.police.uk/advice/personal-safety-and-possessions/dementia-awareness/dementia-awareness/herbert-protocol-missing-person-incident-form>  Leaving information for emergency services professionals: <https://www.neas.nhs.uk/patient-info/message-in-a-bottle.aspx> |  |

[Return to Domain 12](#_Domain_12:_Planning) | [Return to overview](#_top)

# **Information to be gathered at review**

This section outlines the basic information about the patient and carer (where relevant) that should be collated as part of the review, and practical tasks relating to arranging the review and summarising actions in a care plan.

## **Patient details**

| Name |
| --- |
| Preferred name |
| DOB |
| Marital status |
| NHS number |
| GP, surgery |
| Address |
| Access to home in an emergency (door keys and codes) |
| Telephone number |
| Email address |
| Main language |
| Preferred method of communication (including contact method, need for interpreter) |
| Religion |
| Is the patient themselves a carer? |
| Anyone the patient would like to be at reviews/appointment |
| Patient consent given to contact carer about care |
| Information sharing agreement |

## **Carer details**

| Carer consent for their details to be held on patient record |
| --- |
| Name of carer |
| Relationship of carer to patient |
| Address |
| Telephone |
| Email address |
| Preferred method of contact |
| Registered with your practice?  IF YES: On carers register?  IF NO: encourage carer to inform their own GP of patient’s diagnosis and register as a carer |
| Additional people involved in informal care (eg other family members, friends, neighbours) |
| Details of informal care |
| Next of kin or emergency contact (if different from carer) |

## **Current ACP documentation with dates**

|  |
| --- |
| Lasting power of attorney – health & welfare |
| Lasting power of attorney – property & finance |
| DNACPR |
| EHCP |

## **Current immunisations for person with dementia & carer (if registered)**

|  |
| --- |
| Flu vaccine |
| COVID-19 (check number of doses, booster) |
| Tetanus |
| Pneumonia |

[Return to overview](#_top)

# References

This annual dementia review guide was informed by a number of existing templates, as well as the components produced as part of PriDem WS2, review/care planning documents collected at WS2 sites, and published articles.

**Templates consulted**

Ardens dementia template (<https://support-ew.ardens.org.uk/support/solutions/articles/31000161282-dementia>)

Ardens social prescribing template (<https://support.ardens.org.uk/support/solutions/articles/31000154092-social-prescribing>)

Bolton Staying Well Check tool (<https://www.england.nhs.uk/wp-content/uploads/2017/11/dg-case-study-staying-well-check-tool.pdf>)

Comprehensive Geriatric Assessment (<https://www.bgs.org.uk/sites/default/files/content/resources/files/2019-02-08/BGS%20Toolkit%20-%20FINAL%20FOR%20WEB_0.pdf>)

Dementia Care and Support Plan (<https://www.southeastclinicalnetworks.nhs.uk/dementia-csplanning-toolkit-bucks/>)

Diamond Lewy Management toolkit (<https://research.ncl.ac.uk/diamondlewy/managementtoolkit/>)

Framework for Annual Review (Johnathan Kaye) (<https://www.england.nhs.uk/wp-content/uploads/2017/11/dg-case-study-framework-for-annual-review.pdf>)

NHS England Dementia: Good Personalised Care and Support Planning [(https://www.england.nhs.uk/wp-content/uploads/2020/02/FINAL-_Update_Dementia-Good-Care-Planning-.pdf](file:///\\campus\dept\ihs\programmes\ahs\pridem\WS4\PriDem%20annual%20review%20&%20care%20planning%20documents\(https:\www.england.nhs.uk\wp-content\uploads\2020\02\FINAL-_Update_Dementia-Good-Care-Planning-.pdf))

NHS England Social Prescribing and Community Based Support (<https://www.england.nhs.uk/wp-content/uploads/2020/06/social-prescribing-summary-guide-updated-june-20.pdf>)

Year of Care dementia template (<https://www.england.nhs.uk/wp-content/uploads/2017/11/dg-case-study-example-best-practice-care-plan-template.pdf>)

Yorkshire and Humber Care Planning Resources (<https://www.england.nhs.uk/wp-content/uploads/2017/11/cg-case-study-example-qof-annual-review-templates.pdf>)

**Bibliography**

Bamford C, Wheatley A, Brunskill G, Booi L, Allan L, et al. (2021) ‘Key components of post-diagnostic support for people with dementia and their carers: A qualitative study’, *PLOS ONE*, 16(12):e0260506. <https://doi.org/10.1371/journal.pone.0260506>

Dow J and Robinson L (2014) ‘Assessment of carers of people with dementia’, *InnovAiT*, 7(4):233–240. <https://doi.org/10.1177/1755738013519785>

Lee L and Molnar F (2017) ‘Driving and dementia: Efficient approach to driving safety concerns in family practice’, *Can Fam Physician,* 63(1):27-31. <https://www.ncbi.nlm.nih.gov/pmc/articles/PMC5257216/>

Lee L, Patel T, Molnar F, Seitz D. (2018) ‘Optimizing medications in older adults with cognitive impairment: Considerations for primary care clinicians’, *Can Fam Physician,* 64(9):646-652. <https://www.ncbi.nlm.nih.gov/pmc/articles/PMC6135130/>

Tsoi K, Chan J, Hirai H, and Wong S (2017) ‘Comparison of diagnostic performance of Two-Question Screen and 15 depression screening instruments for older adults: Systematic review and meta-analysis’, *British Journal of Psychiatry*, 210(4):255-260. <https://doi.org/10.1192/bjp.bp.116.186932>

Appendix 1 ([return to overview](#_top))

What Could Make A Difference?

We look forward to seeing you at your dementia review. It will be helpful for us to focus on the areas that really matter to you.

Take some time to think about your health and wellbeing. What is working well? What isn’t working well? What could help? Write down the things that are important and you would like to discuss.

These are some things people often like to talk about in their dementia review. Circle any areas that you would like to discuss.

| **Physical health & medication** | **Understanding my dementia diagnosis** | **Developing ways of living with dementia** | **Maintaining skills** | **Information I need** |
| --- | --- | --- | --- | --- |
| **My culture or religion** | **Who to contact for advice** | **Support groups or networks** | **Mood & feelings** | **Relationships & intimacy** |
| **Activities, interests, hobbies, holidays** | **Professionals involved in supporting me** | **What to expect** | **Planning for the future** | **Ways to keep independent & safe** |
| **Support for my friends and family** | **Information for my friends and family** | **Feeling heard** | **Finances (e.g. benefits) & legal issues** | **Managing my home** |

My other concerns / questions

Please take your time to complete this leaflet and bring to your upcoming appointment.

Appendix 2 ([return to overview](#_top))

# **My care plan**

This care plan is a summary of my recent review on [*insert date*] which I did/did not attend

## **People who helped with this care plan**

| Family member/friend(s) | | |
| --- | --- | --- |
| Name | Relationship to me | Contact details |
|  |  |  |
|  |  |  |
| Professionals | | |
| Name | Role | Contact details |
|  |  |  |
|  |  |  |
|  |  |  |
|  |  |  |
|  |  |  |
|  |  |  |

## **Who is my named point of contact?**

We agreed that my named point of contact is [*insert name/team and contact details if not involved in review*]

## **What did we discuss in the review?**

|  |
| --- |

## **What did we decide?**

| Actions for me: | |
| --- | --- |
| What | By when? |
|  |  |
|  |  |
|  |  |

| Actions for my family member/friend | |
| --- | --- |
| What | By when? |
|  |  |
|  |  |
|  |  |

| Actions for professionals | | |
| --- | --- | --- |
| Name/role | What | By when? |
|  |  |  |
|  |  |  |
|  |  |  |
|  |  |  |
|  |  |  |

| Anything else we decided: |
| --- |

My next review will be in [*insert number*] months.
