# Supplementary figures and images for: Inside the black box: Refining programme theory in the PriDem dementia care study
Source: PLoS One. 2026 Mar 17;21(3):e0333154. doi: 10.1371/journal.pone.0333154 (PMC12995305; doi:10.1371/journal.pone.0333154)

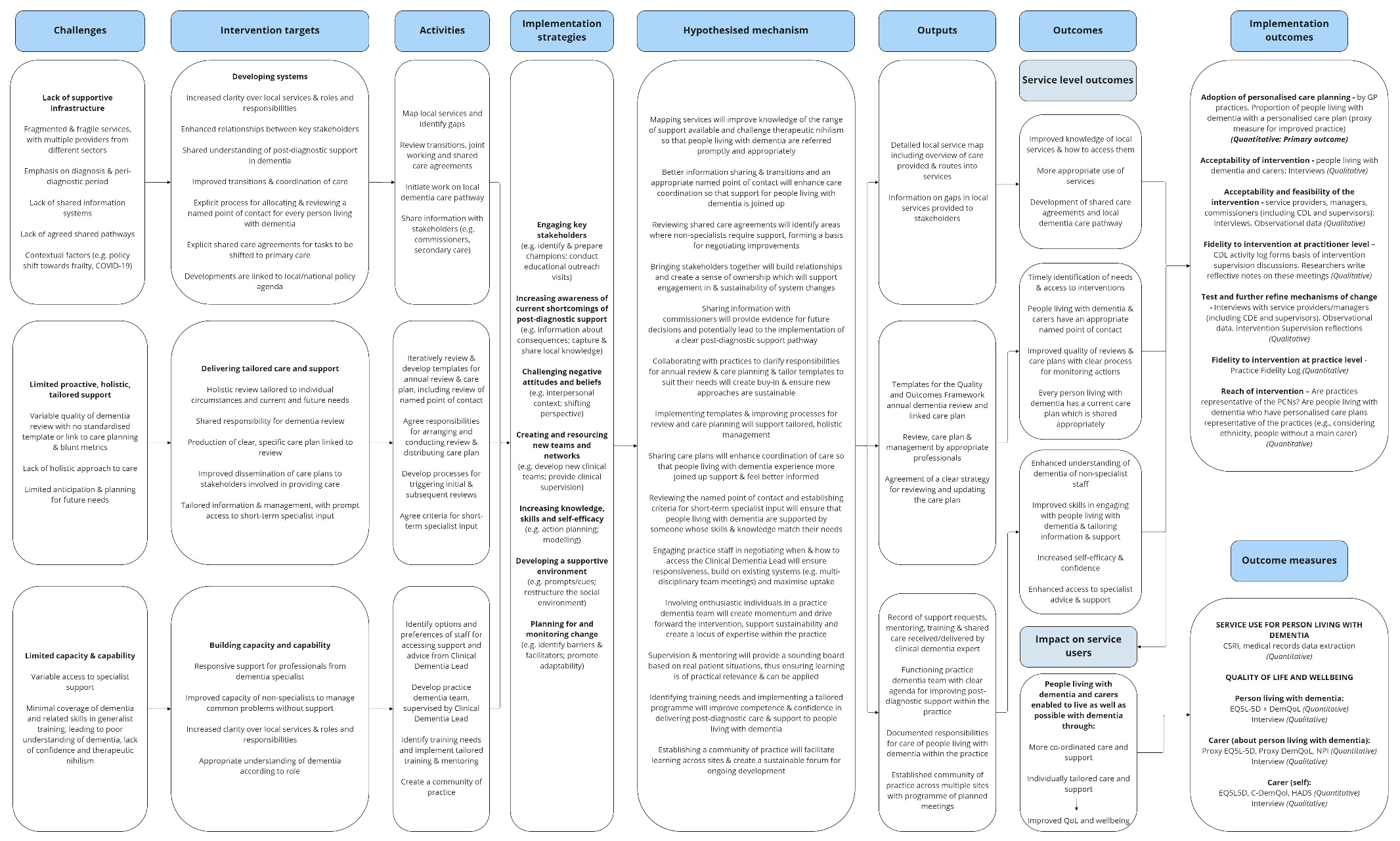

Supplement: S1 Text — S1 File. PriDem Logic Model. S2 File. Standards for Reporting Qualitative Research (SRQR) Checklist. S3 File. Summary of evaluation findings already shared. S4 File. Adaptable PriDem resource pack with review templates. (ZIP) [file pone.0333154.s001.zip › S1 File- PriDem Logic model.docx]
